# Supplementary material for: Identification of photoperiod-induced specific miRNAs in the adrenal glands of Sunite sheep (Ovis aries)
Source: Front Vet Sci. 2022 Jul 22;9:888207. doi: 10.3389/fvets.2022.888207 (PMC9354845; doi:10.3389/fvets.2022.888207)
Supplement: Supplementary file 1 [file Data_Sheet_1.ZIP › Supplementary file/miRDeep2 file.zip!/miRDeep2 file/Know_Structure/LP42A2/oar-mir-154a.pdf]

miRBase precursor : oar-mir-154a
Total read count : 589
oar-miR-154a-5p read count : 8
oar-miR-154a-3p read count : 580
remaining reads : 1

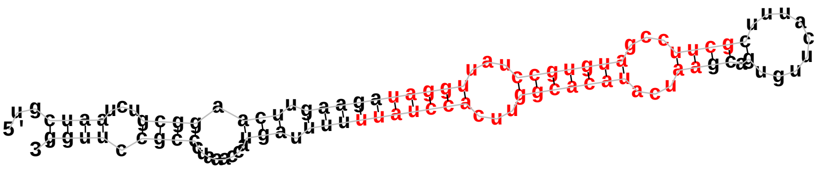

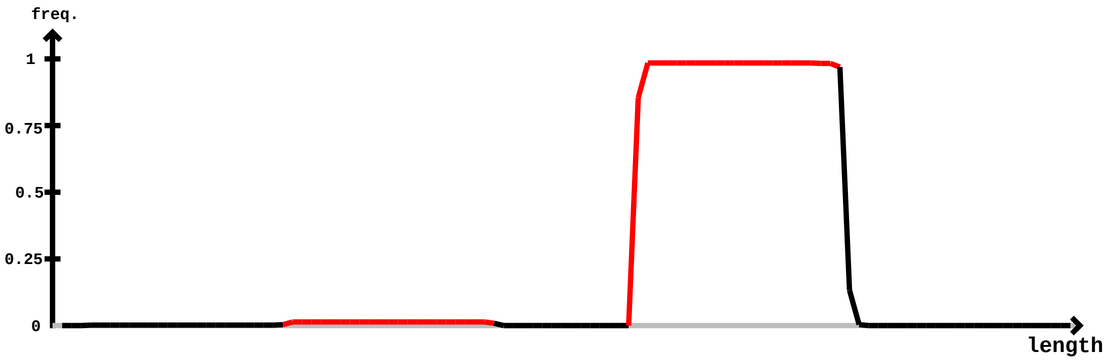

oar-miR-154a-5p

oar-miR-154a-3p

```
5'- ugcuaaucugcggaacuugaagauagguuauccguguagccuucgcuuuacuugugacgaaucauacacgguucaccuauuuuuuaguaccaaaucccgccuugg   -3'    exp
    .((......))((.(((.((((((((((...(((((((...(((((.........).))))...)))))))...)))))))))).))).)).............    reads     mm        sample
    ..cuaaucugcggaacuugaaga..................................................................................    1         0         seq
    ......................auagguuauccguguagccuuc.............................................................    1         0         seq
    .......................uagguuauccguguagccuuc.............................................................    2         0         seq
    .......................uagguuauccguguagccuucg............................................................    5         0         seq
    ...........................................................aaucauacacgguucaccu...........................    1         0         seq
    ...........................................................aaucauacacgguucaccuau.........................    7         0         seq
    ...........................................................aaucauacacgguucaccuauu........................    482       0         seq
    ...........................................................aaucauacacgguucaccuauuu.......................    13        0         seq
    ............................................................aucauacacgguucaccuau.........................    1         0         seq
    ............................................................aucauacacgguucaccuauu........................    11        0         seq
    ............................................................aucauacacgguucaccuauuu.......................    63        0         seq
    ............................................................aucauacacgguucaccuauuuu......................    2         0         seq
```
